# Evaluation of psychological symptoms in patients before and after simultaneous pancreas-kidney transplantation: a single-center cross-sectional study

**DOI:** 10.1590/acb370202

**Published:** 2022-04-22

**Authors:** Thais Malta Romano, Marcelo Moura Linhares, Karin Romano Posegger, Érika Bevillaqua Rangel, Adriano Miziara Gonzalez, Alcides Augusto Salzedas-Netto, Samantha Mucci, Hélio Tedesco Silva-Junior, Gaspar de Jesus Lopes, José Osmar Medina-Pestana

**Affiliations:** 1 Fellow Master degree. Department of Gastroenterology – Postgraduate Program in Interdisciplinary Surgical Science – Universidade Federal de São Paulo – Sao Paulo (SP), Brazil.; 2 Associate Professor. Department of Gastroenterology – Universidade Federal de São Paulo – Sao Paulo (SP), Brazil.; 3 PhD. Department of Nephrology – Universidade Federal de São Paulo – Sao Paulo (SP), Brazil.; 4 PhD. Department of Psychiatry – Universidade Federal de São Paulo – Sao Paulo (SP), Brazil.; 5 Assistant Professor. Department of Nephrology – Universidade Federal de São Paulo – Sao Paulo (SP), Brazil.; 6 Full Professor. Department of Nephrology – Universidade Federal de São Paulo – Sao Paulo (SP), Brazil.

**Keywords:** Anxiety, Depression, Pancreas Transplantation, Kidney Transplantation, Quality of Life

## Abstract

**Purpose:**

Simultaneous pancreas-kidney transplantation (SPKT) brings several benefits for insulin-dependent type-1 diabetic patients associated with end-stage renal disease (ESRD). However, data on psychological outcomes for the waiting list and the transplanted patients are still lacking.

**Methods:**

Using the psychological Beck inventories of anxiety (BAI) and depression (BDI), 39 patients on the waiting list were compared to 88 post-transplanted patients who had undergone SPKT.

**Results:**

Significant differences were found regarding depression (p = 0.003) but not anxiety (p = 0.161), being the pretransplant patients more vulnerable to psychological disorders. Remarkable differences were observed relative to the feeling of punishment (p < 0.001) and suicidal thoughts (p = 0.008) between the groups. It was observed that patients who waited a longer period for the transplant showed more post-transplant anxiety symptoms due to the long treatment burden (p = 0.002).

**Conclusions:**

These results demonstrated the positive impact of SPKT on psychological aspects related to depression when comparing the groups. The high number of stressors in the pretransplant stage impacts more severely the psychosocial condition of the patient.

## Introduction

Type 1 diabetes mellitus is a chronic condition that, if not managed satisfactorily, causes long-term significant complications, including end-stage renal disease (ESRD). Treatment for these patients may include lifestyle change, insulin, and hemodialysis. Although hemodialysis and regular insulin administration provide glycemic control, unfortunately, it significantly decreases the overall quality of life^1^. Patients with severe complications such as advanced nephropathy or ESRD are eligible for kidney or simultaneous pancreas-kidney transplantation (SPKT)^2^.

According to the recommendations of the American Diabetes Association, simultaneous pancreas-kidney transplantation is the best management for type 1 diabetes mellitus patients presenting ESRD^3^. It is considered the gold-standard treatment since it significantly increases the life expectancy of the patient. After transplantation, most patients no longer need insulin and dialysis, thus reversing the established disease^4^.

Despite the transplantation dramatically improves the quality of life, several adaptation difficulties may be present such as psychopathological disorders, adaptation to immunosuppressive drugs, and problems with compliance to treatment protocols. Although depressive symptoms are more likely in patients before the transplantation, the post-transplant period is also critical for the psychological health^5^.

Psychological distress may also influence the course of the main disease. In the pretransplant period, psychological disorders such as depression and anxiety hinder adherence to the treatment and increase the risk of complications such as hypertension, chronic kidney disease, and associated cardiovascular diseases^6^. In fact, depression is still one of the most common symptoms among individuals with ESRD. Approximately 25–30% of patients with ESRD present depressive symptoms, a four times higher rate than the general^5^. It was also seen that negative or positive ways of perceiving cognitively the illness are good predictors of depression in ESRD patients^7^.

Several studies have demonstrated the impact of emotional and psychological distress on patients submitted to heart, lung, isolated kidney, pancreas-kidney transplantation, and others^8^–^10^. Although the quality of life has already been evaluated in SPKT^11^–^13^, the incidence of depression and anxiety has not yet been investigated in such individuals. This study aimed to assess the level of depression and anxiety in patients with type 1 diabetes mellitus and ESRD that are on the waiting list for SPKT transplantation, and in patients after this type of transplant.

## Methods

This study included a total of 127 eligible patients, older than 18 years of age, who accepted to participate in the study and signed an informed consent form. All patients were followed up at the pre- and post-transplantation outpatient clinic of Hospital do Rim at the Universidade Federal de São Paulo.

This work was conducted in accordance with the Declaration of Helsinki of 1975 as revised in 2013, the Declaration of Istanbul 2008, the Brazilian National Health Council Resolution 466/2012, and it was approved by the Ethical Committee of the Universidade Federal de São Paulo, under the number 142892/2018.

### Study design

This cross-sectional, nonrandomized study evaluated 127 patients divided into two groups: pre-SPKT patients waiting on a list for the transplant (n = 39), and post-SPKT individuals (n = 88). The pretransplantation group included insulin-dependent patients with ESRD eligible for transplantation and the post-transplantation group consisted of individuals that had undergone the surgery and had regular follow-up at the hospital. Also, all post-transplantation individuals included in this work passed through the same waiting list at the hospital and therefore were submitted to the same medical evaluation and treatment protocols to the pretransplantation group. Hence the pre- and post-transplantation groups were as homogeneous as possible in terms of primary disease and medical protocols adopted. Clinical parameters as the time of disease, time and type of treatment, and key diabetes-related complications were considered for both groups.

The names of the patients, as well as their telephone numbers, were provided weekly through the pre- and post-transplant outpatient clinics via the integrated computer system. Patients were invited to participate by phone. After accepting, they were interviewed in person in an isolated room in the pre- and post-transplant outpatient clinic from the hospital by trained and prepared interviewers.

### Sociodemographic and psychological evaluation

Psychological evaluation protocols included the Beck Depression Inventory (BDI) and the Beck Anxiety Inventory (BAI)^14^. Both BAI and BDI are 21 items, multiple-choice and self-reported inventories. Each item is scored 0 to 3 points for a total score range of 0 to 63. For BDI, the suggested score ranges for mild depression, moderate to severe depression, and severe depression is 10–18, 19–29, and 31 or above, respectively. For BAI, scores between 10–16 indicate mild anxiety, 17–29 moderate anxiety and scores above 30 represent severe anxiety.

Besides the total score obtained in the inventories, two questions were analyzed independently since they warrant attention as suicide thoughts and negative thoughts about the future. Clinical and demographic data were also collected as well as relevant information about the transplant.

### Statistical analysis

Data analysis was performed using the Statistical Package for Social Sciences (SPSS v18.0, Inc., Chicago IL). Fisher’s exact and chi-square tests were used to compare the frequencies of the 21 variables that compose the two psychological tests (BAI and BDI) between pre- and post-transplant patient groups. Further, two-group comparisons were made using the t-student (parametric) test or the Mann–Whitney test (nonparametric).

## Results

### Sociodemographic characteristics

A total of 127 patients were evaluated, of whom 39 were included in the pretransplant group and 88 were post-transplanted individuals.

The mean age was similar between the groups (36.7 and 35.1 years of age for the pre- and post-SPKT respectively, p = 0.20) assuming the age at the date of the surgery for the post-SPKT group. Significant differences were observed for gender in the pre-SPKT group (male: 28 vs. female: 11; p = 0.022) (Table 1). However, the remaining parameters were comparable, reflecting the homogeneity between the patients included in both groups.

**Table 1:** Demographic and clinical variables for pre- and post-SPKT groups.

Variable			Groups		Total	χ^2^	p
			Pre-SPKT (n = 39)	Post-SPKT (n = 88)			
Gender	Male	N	28	44	72	5.228	0.022*
	Female	N	11	44	55		
Professional Occupation	Working	N	12	29	41	0.059	0.808
	No occupation	N	27	59	86		
Marital Status	Married/committed	N	26	43	69	3.451	0.63
	Single/unmarried	N	13	45	58		
Insulin treated	Yes	N	39	20	59	64.927	< 0.001*
	No	N	0	68	68		
Amputation	Yes	N	2	9	11	0.888	0.345
	No	N	37	79	116		
Vision	Good/satisfactory	N	18	43	61	0.079	0.777
	Bad and blindness	N	21	45	66		

N = total number of patients;

* p < 0.05.

Regarding the time of ESRD, diabetes, and insulin intake (Table 2), the groups presented differences only in the meantime with ESRD (4.68 and 6.83 years) for the pre- and post-transplantation groups respectively (p = 0.009). Predictably, the post-transplanted group has overall more time of disease than the pre-SPKT group, since they also spent more time waiting for the transplantation than several individuals in the pre-SPKT group.

**Table 2:** Description of variables related to the time factor for ESRD, diabetes, and insulin intake.

Variables	Pre-SPKT Group				Post-SPKT Group			U	p
	N	Mean	SD^b^		N	Mean	SD^b^		
Time with known ESRD (years)^c^	39	4.68	2.58		88	6.83	4.98	237.5	0.009*
Time on dialysis (years)^c^	39	2.68	1.9		73	3.57	3.41	277.3	0.833
Time since diabetes diagnosis (years)^c^	39	22.94	5.48		73	24.68	6.60	537.4	0.143
Insulin-treated (years)^c^	39	21.66	7.13		23	22.26	9.9	357.05	0.984
Graft loss after transplant (days)^c^	-	-	-		12	12.9	-	-	-

N = total number of patients;

b SD = Standard Deviation;

c Data obtained at start of study;

* p < 0.05.

### Psychological and anxiety evaluation

The scores obtained on BDI and BAI inventories were compared between the pre- and post-transplant patients (Table 3), and significantly less depression was seen in post-SPKT (p = 0.003). Particularly, marked differences were observed (Table 4) in relation to the symptoms: self-punishment (p < 0.001), suicidal thoughts (p = 0.008), irritation (p = 0.009), feeling tired or having little energy (p = 0.007), alteration in sleep pattern (trouble falling or staying asleep, or sleeping too much) (p = 0.039) and health concerns (p = 0.004).

**Table 3:** Depression and anxiety inventories results for pre- and post-SPKT groups.

Variable	Categories		Groups		Total	P
			Pre-SPKT	Post-SPKT		
BDI	No depression	N	21	70	91	0.003*
	Depression at any level	N	18	18	36	
	Total	N	39	88	127	
BAI	No anxiety	N	23	63	86	0.161
	Anxiety at any level	N	16	25	41	
	Total	N	39	88	127	

N = total number of patients;

* p < 0.05.

**Table 4:** Comparison of the 21 variables related to the BDI psychological test between the pre- and post-SPKT groups.

BDI Symptom	Psychic suffering characterized by depression					χ^2^	p
	Pre-SPKT			Post-SPKT			
	N	%		N	%		
Sadness	1	2.6		4	4.5	0.281	0.596
Pessimism	1	2.6		1	1.1	0.355	0.551
Past Failure	1	2.6		3	3.4	0.063	0.801
Loss of pleasure	1	2.6		1	1.1	0.355	0.551
Guilt	5	12.8		8	9.1	0.409	0.522
Self-punishment	12	30.8		6	6.8	12.744	< 0.001*
Self esteem	1	2.6		0	0.0	2.274	0.132
Self criticism	6	15.4		5	5.7	3.216	0.073
Suicidal Thoughts	3	7.7		0	0.0	6.933	0.008*
Cries	3	7.7		6	6.8	0.031	0.859
Irritation	9	23.1		6	6.8	6.858	0.009*
Loss of Interest	0	0.0		3	3.4	1.362	0.243
Indecision	4	10.3		6	6.8	0.44	0.507
Devaluation / Self Image	5	12.8		6	6.8	1.231	0.267
Loss of energy	14	35.9		13	14.8	7.204	0.007*
Change in Sleep Pattern	2	5.1		17	19.3	4.277	0.039*
Tiredness or Fatigue	4	10.3		4	4.5	1.493	0.222
Appetite Change	1	2.6		1	1.1	0.355	0.551
Weight loss	2	5.1		7	8.0	0.328	0.567
Health Concern	9	23.1		5	5.7	8.337	0.004*
Loss of Interest in Sex	7	17.9		5	5.7	4.753	0.029*

N = total number of patients;

* p < 0.05.

Anxiety (evaluated by the BAI inventory), did not present a significant difference between pre- and post-transplanted patients, though some symptoms were significantly more prevalent among the pre-SPKT group (Table 5), as fear of the worst (p = 0.030), fear of death (p = 0.005), frightening (p = 0.029), and fainting sensation (p = 0.015).

**Table 5:** Comparison of the 21 variables related to the BAI psychological test between the pre- and post-SPKT groups.

BAI Symptom List	Psychic suffering characterized by anxiety					χ^2^	P
	Pre-SPKT			Post-SPKT			
	N	%		N	%		
Numbness or tingling	9	23.1		9	10.2	3.668	0.055
Heat sensation	4	10.3		14	15.9	0.710	0.399
Wobbliness in legs	2	5.1		5	5.7	0.016	0.900
Unable to relax	7	17.9		16	18.2	0.001	0.975
Fear of worst happening	14	35.9		16	18.2	4.701	0.030*
Dizzy / lightheaded	4	10.3		7	8.0	0.181	0.671
Heart pounding / racing	3	7.7		11	12.5	0.637	0.425
Unsteady	5	12.8		8	9.1	0.409	0.522
Afraid / terrified	2	5.1		2	2.3	0.722	0.395
Nervous	13	33.3		19	21.6	1.977	0.160
Feeling of choking	3	7.7		4	4.5	0.514	0.473
Hands trembling	4	10.3		15	17.0	0.979	0.322
Shaking / unsteady	2	5.1		7	8.0	0.328	0.567
Fear of losing control	8	20.5		8	9.1	3.202	0.074
Difficulty in breathing	4	10.3		5	5.7	0.859	0.354
Fear of dying	8	20.5		4	4.5	8.053	0.005*
Scared	7	17.9		5	5.7	4.753	0.029*
Indigestion or discomfort in the abdomen	6	15.4		15	17	0.054	0.816
Feeling faint	4	10.3		1	1.1	5.943	0.015*
Face flushed	2	5.1		4	4.5	0.020	0.886
Hot / Cold sweats	10	25.6		12	13.6	2.719	0.099

N = total number of patients;

* p < 0.05.

It was also evaluated if the time of the ESRD and the time of hemodialysis treatment were related to psychological symptoms. For the pre-SPKT patients, the time of the ESRD was not related to anxiety (p = 0.997) or depression (p = 0.224), as well as the meantime of hemodialysis treatment was not related to the BAI (p = 0.700) nor the BDI (p = 0.614) results. For the post-transplanted individuals, the time of hemodialysis had no impact on depression (p = 0.989) and anxiety (p = 0.658) symptoms. However, the time of ESRD appears to impact the anxiety levels for patients who waited a longer period for the transplant (p = 0.002). This result may be related to the burden caused by a long time under dialysis treatment waiting for the graft. No significant results were obtained for the depressive-related symptoms (p = 0.321) and time of ESRD.

Still, among the transplanted patients, 23 (26.1%) had pancreas graft failure, and, from these, 12 lost the graft (n = 10 pancreas and n = 2 kidney). The mean graft survival after transplantation was 12.9 months. At the time of investigation, 23 patients, who suffered pancreas graft failure or loss, from the post-transplantation group were on insulin (100%), and 2 patients who suffered from kidney graft loss were under dialysis (8.9%). Regardless of the suffering resulting from the loss of function of the graft, those patients requested to be considered for a new SPKT or isolated kidney transplantation. It was not found evidence of emotional alterations in these patients.

In the post-transplant group, there was a clear improvement in the following questions from the BDI: feelings of punishment, irritation, health concern, and interest in sex. And, from the BAI inventory, the most evident improvements were related to the fear of the worst, fear of dying, and being scared.

## Discussion

Chronic ill patients not only present functional and biological limitations but also emotional, cognitive, and social conflicts. Mood and anxiety disorders, for instance, are very common in organ-transplant candidates, being around 25% in patients with severe pulmonary diseases and reaching 50% in patients with advanced cardiac diseases^8^. After transplantation, the psychological burden is expected to be less severe than the preoperative period. Nevertheless, the development of psychiatric disorders in transplanted patients is also relatively common, up to 20% of kidney recipients, an astonishing 63% of heart recipients can suffer from some psychiatric disorder, especially in the first year after surgery^5^.

The present study shows that pre-SPKT individuals have more depression symptoms than the post-SPKT patients, where 46% of patients on the waiting list suffer from depression at some level (mild to severe) but only 20% of post-transplant individuals presented such symptoms. As already reported, the waiting period is painful, stressful, and considered as the most difficult period for patients^15^. Being sick, under dialysis process, and afraid that the transplantation may come too late result in huge stress. Critically, it was demonstrated that higher depression scores are directly related to nonadherence to the treatment in patients that are on the waiting list for kidney transplantation^16^. By analyzing the most significant differences in our study, it is likely that the better results observed in the post-SPKT group were a combination of leaving the waiting list (regardless of the success of the surgery), and the improvement in the quality of life offered by the transplantation^13^. Suicide thoughts and self-punishment for instance were respectively manifested by 8% and 31% of the pre-SPKT group but by 0% and 7% of the post-SPKT, indicating that the transplant was effective in reducing the psychological distress. Interestingly, all post-SPKT patients who lost the graft declared during the interview that they have the interest to be listed again for the transplant. Even with so many risks and with a great number of stressors, the expectation for a life free of disease is superior and may be one of the factors that cause these patients to have lesser psychic suffering.

Although anxiety had not a significant difference between the pre- and post-transplantation patients, some anxiety-related symptoms were significantly more prevalent in pre-SPKT, such as fear of the worst and fear of death. Feeling fainting was also statistically different, but it is difficult to determine the real cause of this symptom. Patients can experience weakness and feel fainting as a consequence of the stress, or because of the primary disease. Diabetes presents many potential pathways for fatigue and weakness encompassing physiological, psychological, and lifestyle factors^17^.

Despite the anxiety and depression are more prevalent in the pre-SPKT group, this study analysis revealed that the presence of anxiety was related to the time of ESRD in the post-transplant group. One would expect that after transplantation, the improvement in life expectancy would mitigate psychological symptoms over time. However, the psychosocial background of SPKT patients is often complicated due to long-standing disease history. These individuals are also subjected to great stressors after the surgery as the side effects of immunosuppressive medications and the limitations imposed by the ESRD as disability, loss of vision, delayed independence of care, and retirement at an early age. Critically, 67% of the post-transplanted patients are unemployed/retired, and, among those with anxiety symptoms, 77% are unemployed or retired. Depressive symptoms and anxiety incidences are high in solid organ transplanted patients^18^. Furthermore, it was reported that depression rates can increase in patients after renal transplantation through the years, from 5.0% to 9.1% in patients after 1 and 3 years of surgery, respectively^19^. It is likely that a similar situation occurs after SPK transplantation, as one of the requirements for the SPKT is the long-standing disease history that already caused irreversible limitations. Therefore, the patients might feel angry or frustrated after the surgery and develop poor coping.

Some important limitations of this study may be considered. Due to the small number of patients, in the pre-SPKT group, it was not possible to correlate all the clinical data to the psychological profile. Also, the study design does not allow longitudinal evaluation of the same patients.

Although not excluded in the analysis of patients who lost the graft or graft function after transplant, an overall improvement of depressive and anxiety symptoms after the transplantation was noticed. In particular, depression symptoms as healthy concern and interest in sex were improved and decreasing in anxiety symptoms as the fear of the worst and fear of death were observed.

The interest in sexual activities was also the subject of a detailed study^19^ in SPKT transplant patients. The authors found that patients that received kidney and pancreas simultaneously reported an overall improvement in erectile function in comparison with kidney-only transplantation and ESRD patients.

Although the quality of life in SPKT patients was more extensively explored, while conducting this study, no studies on depression and anxiety performed in diabetic patients with ESKD comparing pre- and post-SPK transplantation patients were identified in the literature^12^^,^ 13 ^,^ 20–23. A previous study described significant improvement in the quality of life of transplanted patients from the same cohort, sustaining that the transplant event brings positive feelings, decreases negative moods ultimately leading to psychological improvement^12^.

The psychosocial condition of SPKT individuals is complicated because of the number of stressors. Among many consequences related to diabetes mellitus and ESRD, multiple losses are experienced by the patient as restricted diet, hemodialysis (which results in loss of autonomy, and self-esteem), and, sometimes, financial issues contribute to the incidence of psychosocial morbidity. Although the number of stressors is usually higher in the pretransplant stage when the patient faces limited life expectancy and limited autonomy, it is not uncommon for the patients to experience anxiety even after the transplantation. Given these factors, appropriate psychological and emotional support might be essential to better assist patients in the pre- and post-transplantation process^24^.

## Conclusions

When comparing post-SPKT to pre-SPKT patients, some psychosocial symptoms such as depression, suicide thoughts, guilty, and irritation were observed and required additional attention. The emotional and physical distress of long-term treatment, medical exams, and the uncertainties related to the future transplant are factors that lead patients to loss of hope and quality of life. Early identification of psychological and psychiatric symptoms and further psychotherapeutic interventions in patients candidates to SPKT may support them throughout the transplantation waiting process as well as to a better post-transplant outcome.
