# ERRATA: Evaluation of psychological symptoms in patients before and after simultaneous pancreas-kidney transplantation: a single-center cross-sectional study

**DOI:** 10.1590/acb370202.errata

**Published:** 2022-05-23

In the manuscript **Evaluation of psychological symptoms in patients before and after simultaneous pancreas-kidney transplantation: a single-center cross-sectional study**, which DOI is: https://doi.org/10.1590/acb370202, published in the journal **Acta Cirúrgica Brasileira**, 2022;37(02)e370202, page 1:

**Instead of**:

Érika Bevillaqua Rangel^3^

3. PhD. Department of Nephrology – Universidade Federal de São Paulo – Sao Paulo (SP), Brazil.

**Should be**:

Érika Bevilaqua Rangel^3^

3. Assistant Professor. Department of Medicine – Universidade Federal de São Paulo – Sao Paulo (SP), Brazil.

